# Correspondence

**Published:** 1868-10-15

**Authors:** B. S. Woodworth


### *CORRESPONDENCE.*

*Editor* Chicago Medical Journal:

Sir: I read a communication in the September number of your Journal, from B. F. Lightfoot, M.D., of Murraysville, Ill., which gave an account of a case of so-called colic, and, as the doctor "regrets the views of some of the numerous readers of the Journal," I respectfully ask leave to make a few comments on this *remarkable* case. As nothing is said of the previous history of the case, (except that a Dr. G. had given the patient some *Podophyllin*, which operated drastically), nothing of the diathesis, temperament, habits, whether strumous or otherwise, we can not so *positively* infer that the extraordinary treatment was the direct cause of the fatal result, as we obviously might, if we knew that this boy was not one of the strongest and most robust subjects. I wonder it never entered the head of Dr. Lightfoot that the immense quantities of powerful drugs that this boy took, had any thing to do with the grave symptoms which his patient exhibited after he fell into his hands. I should certainly expect that if I treated a patient in that terribly heroic way, whatever the disease might be, (or even if he had *no* disease) that he would speedily become worse, and, unless he had the constitution of a mule, he would inevitably succumb.

I think it would puzzle Dr. Lightfoot himself to show what were the indications, for almost any of the powerful drugs which he exhibited to this boy, to say nothing about the extraordinary doses — and I wish to say here *en passant*, that when a country doctor deals out his own medicine at the bedside, *he is very likely to give twice as much as he professes*, (or thinks he is giving) *to give.* This is my opinion, judging from what I saw in my younger days. Only think of it — a boy of sixteen years, takes, for several days, ten grain doses of *Calomel*, and three and one-half grains Dover powders every four hours, then *Iodide of potassium*, (in doses we are not informed,) *Syrup of iodide iron*, opiates, astringents, tonics, etc., etc., and all for what? Why, for what he called a tumor! It would seem that the boy had tympanitis, for the doctor says it (the swelling) commenced near the "center of the abdomen" and extended into the right iliac region. Not satisfied with the great irritation which the *Podophyllin* had induced, "with the copious watery discharges," he goes on to give him more irritants, such as *Calomel*, *Iodide potassium*, *Iodide iron*, etc., etc. If this boy did not have peritonitis (perhaps subacute) when he commenced his treatment, I don't see how it could be otherwise than developed soon after by the monstrous quantities of drugs exhibited, and it is no wonder to me that the patient "was a mere skeleton at his death." As no *post-mortem* examination was made, we shall never know what ravages these drugs made.

I *had* supposed, before I read this paper of Dr. Lightfoot's, that this *excessive* over-dosing system was about obsolete, even in the most benighted rural districts of this country, but it seems I was mistaken.

We ought, however, to thank Dr. Lightfoot for his account of this case, and it ought to be held up as a warning against a system which, to say the least, is a mere routine of guesswork, random course. This case shows, also, how long a patient may withstand a course of drugs, which, to use as mild a term as deserved, is damnable. *I* consider it barbarous. It would do no discredit to the doctors who treated Charles the II., *vide* Macauley. It is just this sort of therapeutics that has made believers in that miserable delusion, homœopathy, and if we ever expect to make any headway against this, as well as divers other impositions upon the public credulity, we must have a more rational system, *more generally* practiced by us so-called regulars.

I would advise our brother to read Flint, Aitken, Bennett, or Reynold's System of Medicine, (to say nothing about

"Forbes Nature and Art," Bigelow *et al.*) and no longer trust to such obsolete books as Eberle's Practice, and he will never be guilty of treating a patient in that way again.

B. S. Woodworth, M.D.

